# Grape Polyphenols Increase the Activity of HDL Enzymes in Old and Obese Rats

**DOI:** 10.1155/2013/593761

**Published:** 2013-07-14

**Authors:** Andriy L. Zagayko, Ganna B. Kravchenko, Oksana A. Krasilnikova, Yuri O. Ogai

**Affiliations:** Biochemistry Department, National University of Pharmacy, Melnikova street, 12, Kharkiv 61002, Ukraine

## Abstract

HDL particles are protein-rich particles that act as a vehicle for reverse cholesterol transport from tissues to the liver. The purpose of this study was to investigate age-dependent changes in the functional activity of HDL and the effect of high-energy diet on this index, as well as to correct it under the influence of grape polyphenols from “Enoant” obtained from *Vitis vinifera* grapes. We observed the age-dependent composition changes in HDL particle. It was shown that total lipids and triacylglycerol (TG) levels were higher in 24-month-old animals. In obese rats, HDL total lipids and TG levels were higher in 24-month-old than in the 3-month-old and 12-month-old groups but did not differ from 24-month-old group. The plasma HDL paraoxonase (PON) and lecithin:cholesterol acyltransferase (LCAT) activity levels were decreased in old-aged rats, and cholesteryl ester transfer protein (CETP) activity was higher in old rats. Keeping 12-month-old animals on high-fructose diet completely leveled the age differences in the data that have been measured between 12-month-old and 24-month-old rats. After “Enoant” administration, an increase of HDL PON and LCAT activity levels and a reduction of CETP activity were found in 24-month-old and obese rats.

## 1. Introduction

Coronary vascular diseases (CVDs) are the leading cause of morbidity and mortality in adults [1]. Major risk factors for CVD in addition to a sedentary lifestyle and a diet high in saturated fat and cholesterol include family history, age, sex, elevated low-density-lipoprotein cholesterol (LDL-C), decreased high-density-lipoprotein cholesterol (HDL-C), hypertension, cigarette smoking, and diabetes mellitus [2–4].

HDLs are synthesized in the liver and intestine and are responsible for transporting 20–30% of the total plasma cholesterol. Apolipoprotein A-I is the most abundant protein in HDL. The remaining protein mass is made up of minor amphipathic proteins, such as apoC, apoE, apoD, apoM and apoA-IV, enzymes, such as paraoxonase (PON) 1 and platelet-activating factor acetylhydrolase, and glutathionehlt peroxidase, and lipid transfer proteins, such as lecithin : cholesterol acyltransferase (LCAT) and cholesteryl ester transfer protein (CETP) [5].

CETP is physically associated with HDL particles and facilitates the transport of cholesteryl ester from HDL to apoB-containing lipoproteins. A decrease of CETP activity may increase HDL-C and decrease VLDL-C and LDL-C. Thus, CETP simultaneously affects the composition and concentration of apoA−/− and apoB-containing lipoproteins. Clinical studies demonstrate a low prevalence of coronary heart disease among subjects with CETP deficiency [6]. Although CETP deficiency might prevent atherogenesis by increasing HDL-cholesterol levels, their effect has been controversial. Studies performed in Japan indicate that CETP deficiency might increase cardiovascular disease risks [7]. Davidson et al. reported on a CETP vaccine that induces autoantibodies that specifically bind and inhibit endogenous CETP, with the intention of increasing HDL and reducing the development of atherosclerosis [8]. LCAT is a key enzyme necessary for extracellular cholesterol metabolism [9]. LCAT may facilitate the uptake of cholesterol from peripheral tissues into HDL particles by maintaining a concentration gradient for the efflux of free cholesterol [10]. If LCAT is impaired, mature HDL generation would presumably be decreased, resulting in augmentation of atherosclerosis [11].

There are data suggesting that the direct antioxidant effect of HDL on LDL oxidation, measured as a reduction in lipid peroxides, is likely mediated by PON1 [12]. High serum cholesterol and insulin resistance are, for example, associated with decreased PON1 activity [12]. Aging is associated with alteration PON1 activities as a consequence of a higher content of PON1 molecules in the blood, which probably corresponds to the need to respond to the negative effects of senescence. PON1 decreases in older people and with the beginning of menopause [13].

Serum PON1 activity and concentration have also been shown to be modulated by lifestyle and dietary factors [14]. Polyphenols (present in wine, tea, and fruit juice) also increase PON1 activity in both humans and mice [15, 16].

In earlier, studies it was shown that consumption of red wine or its polyphenols quercetin or catechin by apolipoprotein E-deficient mice (whose plasma PON1 activity is lower than controls), was associated with an increase in serum PON activity [17]. Administration of a mixture of red wine polyphenols increased hepatic PON1 activity in mice, while a higher dosage levels had an opposite effect [18]. The low dose of polyphenols was also capable of reversing the decrease of plasma and hepatic PON1 activities and of liver mRNA levels present in mice with hyperhomocysteinemia.

One of the richest sources of polyphenols is *Vitis vinifera,* and products of its processing, including flavonoids and other polyphenols of grape, wine, and grape seeds, are of a great interest due to their antioxidant properties and the ability to scavenge free radicals [19].

Studies in vitro have shown that grape products inhibit the oxidation of LDL. The activity of those substances as oxidation inhibitors in wine diluted 1,000 times markedly exceeded the analogous values for vitamins C and E [20]. In our research, we have used polyphenolic concentrate “Enoant” obtained from *Vitis vinifera* grapes and manufactured by Ressfood Company (Yalta, Ukraine).

The purpose of this study was to investigate age-dependent changes in the functional activity of HDL and the effect of high-energy diet on this index, as well as a way to correct it under the influence of grape polyphenols from “Enoant.”

## 2. Materials and Methods

### 2.1. Animals

3-, 12-, and 24-month-old Wistar male rats which had a free access to food and water and were kept at 24°C on a cycle of 12 h light/12 h darkness were used for experiments. Polyphenol concentrate was given os daily during 21 day (at a dose of 9 mg in recalculation on polyphenols/100 g body weight) [21]. Control animals were given the corresponding volume of physiological solution.

Rats from “Obese” group, 12-month-old rat, were kept on a diet that consisted of 60% fructose. Both diets contained equal percentage of carbohydrates (70%), proteins (20%), and lipids (10%) [22].

The animals were decapitated under chloralose-urethane anesthesia. All procedures were approved by National University of Pharmacy Institutional Animal Care and Use Committee and are in accordance with PHS guidelines.

### 2.2. Isolation of HDL

HDL (*d* 1.085–1.190 g/mL) was isolated from the rat blood plasma by sequential ultracentrifugation and was washed at its lower density limit. Lipoproteins were extensively dialyzed against 10 mM Tris-HC1 buffer (pH 7.4), containing 150 mM NaCl, 0.01% (w/v) sodium azide, and 0.25 mM EDTA (Tris-NaC1 buffer). To distribute the plasma lipoproteins, samples were centrifuged at 65,000 rpm (342,000 g) for 4 h at 4°C in an Optima XL-100 K ultracentrifuge (Beckman Coulter) set at slow acceleration and deceleration. Samples were fractionated within 1 h of centrifugation. 

### 2.3. Determination of Lipid Levels

Lipids were extracted with chloroform and methanol (1 : 2 v/v) 2 times as described [23], and the supernatant were collected for the determination of triglycerides (TG) and cholesterol. TG and cholesterol were determined by enzymatic colorimetric methods with commercial kits (Zhongsheng, Beijing, China). Total cholesterol content was detected with the help of standard enzymatic cholesterol oxidase kits (Biocon Diagnostik GmbH, Germany). Total lipid concentration was determined with the help of standard kit (Eagle Diagnostics, USA) reaction with the vanillin reagent.

### 2.4. Determination of Lipid Peroxide Product Quantity

Lipids from various samples were extracted by chloroform : methanol (2 : 1, v/v) [23]. Lipids in the chloroform phase were recovered by evaporating the chloroform under oxygen-free N_2_ (ultrapure carrier, Grade 99.99% N_2_). The final total lipid extract was dissolved in cyclohexane (1 mg/mL) and then scanned against a cyclohexane blank in a Beckman Model 25 spectrophotometer. Optical density was measured at wavelength of 220 nm (for compounds with isolated double bonds), 232 nm (for diene conjugates), and 278 nm—for ketodienes and conjugated trienes. 

### 2.5. Measuring of *α*-Tocopherol

A modified version of the high-performance liquid chromatography (HPLC) procedure [24] was used to measure vitamins E. The HPLC system included a 150 × 3.9 mm Nova-Pak C18 (4 microns) column with a Guard-Pak precolumn (both from Waters, Milford, MA, USA), Waters Millipore TCM column heater, Waters 490 multiwavelength detector, Hitachi 655-61 processor, Hitachi 655A-11 liquid chromatography, and Bio-Rad autosampler AS-100. 

### 2.6. Assay of PON1

PON1 activity was assayed using synthetic paraoxon (diethyl-p-nitrophenyl phosphate) as substrate. PON1 activity was determined by measuring the initial rate of substrate hydrolysis to p-nitrophenol, which absorbance was monitored at 412 nm in the assay mixture containing 2.0 mM paraoxon, 2 mM CaCl_2_, and 20 *μ*L of plasma in 100 mM Tris-HCI buffer (pH 8.0). The blank sample containing incubation mixture without plasma was run simultaneously to correct for spontaneous substrate breakdown. The enzyme activity was calculated from E412 of p-nitrophenol (18.290 perM/cm) and was expressed as U/mL [25].

### 2.7. CETP Activity

CETP activity in duplicate 10 *μ*L aliquots of plasma was determined after incubations with ^3^H-cholesterol ester-labeled HDL_3_ and LDL. Radioactivity transferred from ^3^H-HDL_3_ to LDL (measured in the supernatant after precipitation with heparin/MnCl^2+^) was used to calculate CETP activity (expressed as the percentage of radioactivity transferred from ^3^H-HDL_3_ to LDL per 16 h of incubation) [26]. 

### 2.8. LCAT Activity

LCAT activity measured by determination of the amount of radioactivity in each spot permits the calculation of the free cholesterol/total cholesterol ratio in each plasma sample before and after the LCAT reaction and thus the estimation of the esterification rate [27]. The fractional esterification rate (%, h′) expressed the percentage of free cholesterol esterified in the plasma sample per hour.

### 2.9. Statistical Analysis

The differences between the results were evaluated using analysis of variance (ANOVA) and Student's *t*-test. In all of the analyses, a difference of *P* < 0.05 was considered statistically significant. The data are expressed as the means ± standard deviations.

## 3. Results

Table 1 shows the plasma lipid and apolipoprotein concentrations of the experimental animals. Total cholesterol level was higher in 24-month-old than in the 3-month-old and 12-month-old groups. The cholesterol level was greater in the obese rats than in the middle-aged and young animals (*P* < 0.05) but did not differ between the 3-month-old and 12-month-old groups. Triglycerides, apo A1, and apo B levels were not different among these three groups. HDL level was higher in 24-month-old than in the 3-month-old and 12-month-old groups. LDL level was higher in the obese rats (*P* < 0.05).

In our experiment, we also observed that the age-dependent composition changes in HDL particle (Table 2). It was shown that total lipids and TG levels were higher in 24-month-old than in the 3-month-old and 12-month-old groups. In obese rats, HDL total lipids and TG levels were higher in 24-month-old than in the 3-month-old and 12-month-old groups but did not differ between 24-month-old group. Total HDL cholesterol level was significantly lower in 24-month-old and obese animals than in the 3-month-old and 12-month-old groups. There was no difference between old-aged and obese rats. It was shown that diene conjugates, ketodienes + conjugated, and total hydroperoxides levels were higher in 24-month-old than in the 3-month-old and 12-month-old groups. Isolated double bonds level was significantly lower in old-aged and obese rats.

Table 3 shows the plasma HDL PON, LCAT, and CETP activity in experimental rats of different age and obese rats. The plasma HDL PON and LCAT activity levels were decreased in old-aged rats, and CETP activity level was higher in old rats.

**Figure 1:**
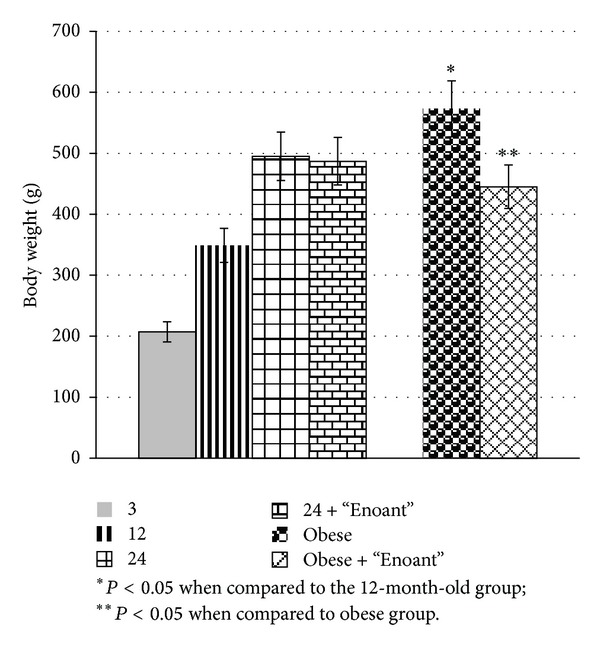
The effect of “Enoant” on the body weight of investigated rats, in each group *n* = 10.

Figure 1 shows body weight of different ages and obesity groups of experimental animals. These data suggest that keeping rats on a fructose-rich diet resulted in a significant increase in body weight. “Enoant” administration decreased this index, which, however, did not reach the control values.

“Enoant” administration decreased HDL diene conjugates, ketodienes + conjugated, and total hydroperoxides levels in 24-month-old and obese rats groups and increased HDL total lipids and TG levels (Table 4). It was shown that *α*-tocopherol level was significantly higher in HLD under “Enoant” administration.

The effect of “Enoant” on the plasma HDL PON, LCAT, and CETP activity levels in experimental is shown in Table 4. After “Enoant” administration, it was found an increase of HDL PON and LCAT activity levels and a reduction of CETP activity in 24-month-old and obese rats.

## 4. Discussion

We have carried out a series of experiments to study the age-dependent changes in the functional activity of HDL, the effect of high-energy diet on this index.

We found that in older animals, there was a significant increase in both HDL and LDL (Table 1). The increase of lipid peroxidation and the reduction of endogenous antioxidant *α*-tocopherol level and the increase of TG levels and key enzymes of HDL activity (Tables 2 and 3) could indicate that the functional activity of HDL in old animals is lower than that in younger ones. 

Data from the Prospective Cardiovascular Münster (PROCAM) Study have shown that having a low HDL cholesterol level was the predominant characteristic of subjects older than 60 years with a history of myocardial infarction compared to subjects older than 60 years without previous cardiovascular events [28].

The HDL fraction is composed of heterogeneous particles with sizes ranging from 7 to 14 nm [2, 12]. In addition to its roles in reverse cholesterol transport and cholesterol esterification, HDL has antioxidant activity that is mostly due to its association with PON1 and anti-inflammatory, antithrombotic, and vasodilation activities that may account for the antiatherogenic action of the lipoprotein [12, 13].

Currently, in the literature, there are contradictory data on the content and activity of PON age-dependent changes [28, 29]. While a major determinant of PON1 activity is represented by genetic polymorphisms, additional factors, not discussed in this paper, should also be mentioned. Age plays the most relevant role, as PON1 activity is very low before birth and gradually increases during the first year or two of life in humans [30]. PON1 activity may also decline with aging, possibly because of the development of oxidative stress conditions [31].

The inhibition of LDL oxidation is a major antiatherogenic property of HDL [32]. This activity is, partially due, to HDL associated proteins and enzymes. However, whether these proteins interact in the antioxidant activity of HDL is unknown. In an impressive study conducted to understand this situation, LDL was incubated with apolipoprotein A1, LCAT, and PON1 in the presence or absence of HDL under oxidizing conditions. When LDL lipid peroxide concentrations were determined, ApoA-1, LCAT, and PON1 were all found to inhibit LDL oxidation in the absence of HDL and enhance the ability of HDL to inhibit LDL oxidation [33].

Age-related loss of PON activity was not found in elderly patients, which, however, associated with the heterogeneity of patient population [34]. It is known that one of the main functions of PON is to protect the HDL from lipid peroxidation processes. In this study, we found a correlation between the decrease in PON activity (Table 3) and an increase in lipid peroxidation of HDL (Table 4).

Thus, a decrease in LCAT activity in 24-month-old animals may be one of the causes of increased oxidized LDL levels in old age [35], which in turn, may increase the risk of cardiovascular disease.

One of the major cardiovascular risk factors is obesity. Keeping 12-month-old animals on a high-fructose diet is accompanied not only by an increase in body weight and lipid metabolism disorders, but also the exchange of lipoproteins. It should also be noted that high-calorie diet completely eliminates the age differences between the studied parameters in 12-month-old animals with obesity and 24-month-old animals. Thus, animals with obesity tend to have significantly more intense lipid peroxidation, lower levels of antioxidants, and reduced activity levels of LCAT and PON.

Previous researches in our laboratory using experimental model of metabolic syndrome have shown that the introduction to animals of grape polyphenols complex inhibited LDL oxidation, HDL lipid peroxidation, normalized PON and CETP activity levels [36].

Therefore, the next stage of our research was to study the influence of grape polyphenols on the composition and functional state of HDL. We have found that “Enoant” significantly inhibits peroxidation processes in HDL and increases the activity of HDL enzymes that indicates an improvement in the functional activity of HDL (Table 4).

PON-1 has also been shown to protect against CVD: (a) it prevents the formation of oxidized HDLs and low-density lipoproteins (LDLs) [12]; (b) it hydrolyses the thiolactone form of homocysteine, which alters proteins in the arterial wall [37]; and (c) it hydrolyses platelet-activating factor, a bioactive phospholipid which is involved in vascular disease development [38]. Several studies suggest that a low-level plasma PON-1 activity is associated with increased prevalence of atherosclerosis and could be an independent risk factor for coronary events [12, 39]. 

Several dietary polyphenols, and in particularly quercetin, have been shown to upregulate PON1 [15]. Resveratrol, a polyphenolic phytoalexin found in grapes and wine, was shown to increase PON1 gene expression in human hepatocyte primary cultures and in HuH7 cells [40]. This effect appeared to be dependent upon activation of the Ah receptor and to involve an XRE-like element in PON1's promoter within the −126 and −106 region. Grape seed extracts have also been reported to increase serum PON activity in control, and particularly in streptozotocin-induced diabetic rats [41]. Polyphenols may upregulate PON1 expression and release by sequential activation of protein kinase A and PPAR-*γ* [42]. Except these, quercetin administration to mice lacking the LDL receptor (LDL−/−) for four weeks was reported to increase liver PON1 mRNA and serum PON1 activity by 40–90%, depending on the dose [16]. 

## 5. Conclusions

Thus, results of our investigations showed an age-dependent increase in HDL lipid peroxidation and a decrease in the PON activity in obese and 24-month-old animals, which may indicate a decrease of the functional activity of HDL. Keeping 12-month-old animals on high-fructose diet completely leveled age differences in the test indices between 12-month-old rats and 24-month-old rats. “Enoant” administration restored blood indices in rats with obesity and reduced peroxidation processes and normalized the PON and LCAT activity levels in old rats.

## Figures and Tables

**Table 1:** Plasma biochemical parameters of the investigated rats, in each group *n* = 10.

Parameters	3 months	12 months	24 months	Obese
HDL (mg/dL)	41.56 ± 6.37	43.49 ± 7.19	50.67 ± 3.15*	52.99 ± 6.31*
LDL (mg/dL)	95.66 ± 7.29	102.45 ± 7.93	104.53 ± 9.78	143.84 ± 10.11*
Cholesterol (mg/dL)	113.56 ± 9.96	155.67 ± 4.33	190.78 ± 9.87*	201.93 ± 15.13*
TG (mg/dL)	86.45 ± 1.56	100.83 ± 2.08	101 ± 3.56	119 ± 4.61*
Apo A1 (mg/dL)	128.56 ± 3.76	135.67 ± 1.89	141.28 ± 3.78	131.56 ± 4.12
Apo B (mg/dL)	70.56 ± 1.82	88.23 ± 2.57	80.36 ± 1.93	87.89 ± 4.32

The data are expressed as means ± SD; *P < 0.05 when compared to the young and middle-aged groups.

**Table 2:** The plasma HDL composition of the investigated rats, in each group *n* = 10.

Parameters	Group			
	3 months	12 months	24 months	Obese
Total lipids, % of the total HDL composition	49.45 ± 1.35	51.45 ± 2.32	57.31 ± 1.93*	55.31 ± 1.93*
Total cholesterol, % of the total HDL composition	14.97 ± 0.23	14.56 ± 0.41	11.21 ± 0.76*	10.23 ± 0.66*
TG, % of the total HDL composition	1.75 ± 0.07	2.15 ± 0.08	2.75 ± 0.14*	2.98 ± 0.15*
*α*-Tocopherol, mmol/L	8.02 ± 0.39	7.88 ± 0.49	6.71 ± 0.45*	5.70 ± 0.49*
Isolated double bonds, U/mL	8.64 ± 0.59	8.33 ± 0.58	7.31 ± 0.17*	7.48 ± 0.25*
Diene conjugates, mmol/L	18.88 ± 1.14	21.78 ± 2.10	29.63 ± 2.35*	31.05 ± 1.65*
Ketodienes + conjugated trienes, U/mL	1.15 ± 0.08	1.21 ± 0.07	1.38 ± 0.06*	1.39 ± 0.07*
Total hydroperoxides, mmol/L	69.04 ± 2.88	69.37 ± 3.46	73.31 ± 1.57*	74.31 ± 1.43*

The data are presented as mean ± SD or percentage.

*P ≤ 0.05 versus intact 3-month-old animals.

**Table 3:** Plasma HDL PON, LCAT, and CETP activity levels in the investigated rats, in each group *n* = 10.

	3 months	12 months	24 months	Obese
PON 1 (nmol min^−1^ mL^−1^)	97.42 ± 4.42	85.15 ± 4.01	71.45 ± 2.21*	76.23 ± 3.12**
LCAT, mkmol/L/h	54.92 ± 1.58	44.86 ± 1.99	36.25 ± 1.28*	20.25 ± 2.28**
CETP, mkmol/L/h	20.42 ± 1.76	33.51 ± 1.98	68.32 ± 8.54*	86.88 ± 9.43**

The data are expressed as means ± SD; *P < 0.05 when compared to the young and middle-aged groups; **P < 0.05 when compared to 24-month-old group.

**Table 4:** The effect of “Enoant” on the plasma HDL composition and the plasma HDL paraoxonase, LCAT, and CETP activity levels in male 24-month-old and obese rat groups, in each group *n* = 10.

Parameter	Group	
	24-month-old + “Enoant”	Obese + “Enoant”
Total lipids, % of the total ApoB-LP composition	52.01 ± 1.87*	52.34 ± 1.88**
Total cholesterol, % of the total ApoB-LP composition	13.34 ± 0.79*	12.56 ± 0.673**
TAG, % of the total ApoB-LP composition	2.05 ± 0.21*	1.98 ± 0.31**
*α*-Tocopherol, mmol/L	7.15 ± 0.55*	6.70 ± 0.52*
Isolated double bonds, U/mL	7.83 ± 0.27*	7.99 ± 0.31**
Diene conjugates, mmol/L	22.63 ± 1.55*	22.54 ± 1.85**
Ketodienes + conjugated trienes, U/mL	1.28 ± 0.08*	1.25 ± 0.09**
Total hydroperoxides, mmol/L	70.32 ± 1.47*	71.54 ± 1.33**
PON 1 (nmol min^−1^ mL^−1^)	79.31 ± 3.29*	81.64 ± 3.04**
LCAT, mkmol/L/h	33.81 ± 1.95*	42.04 ± 3.57**
CETP, mkmol/L/h	58.52 ± 4.13*	77.88 ± 5.64**

The data are expressed as the means ± SD; *P < 0.05 when compared to 24-month-old group; **P < 0.05 when compared to obese group.
